# From Lung Cancer Predictive Models to *MULTIPREVENTion*

**DOI:** 10.3390/jcm15020629

**Published:** 2026-01-13

**Authors:** Zuzanna Budzińska, Zofia Budzisz, Marta Bednarek, Joanna Bidzińska

**Affiliations:** 1 Faculty of Health Sciences with the Institute of Maritime and Tropical Medicine, Medical University of Gdańsk, 80-210 Gdańsk, Poland; z.budzinska@gumed.edu.pl (Z.B.); z.budzisz@gumed.edu.pl (Z.B.); 2 Second Department of Radiology, Medical University of Gdańsk, 80-210 Gdańsk, Poland; marta.bednarek@gumed.edu.pl

**Keywords:** low-dose computed tomography, predictive model, screening, lung cancer, multimorbidity

## Abstract

The early diagnosis and treatment of civilizational diseases remain a significant challenge worldwide. Although advances in medical technology have led to the introduction of more screening options over time, these measures are still insufficient to effectively reduce mortality from deadly diseases such as lung cancer (LC), cardiovascular diseases (CVD), diabetes, and chronic obstructive pulmonary disease (COPD). These conditions pose a major public health burden, underlying the urgent need for more comprehensive and efficient prevention strategies. Recently, the concept of ‘multiscreening’ has emerged as a promising approach. Multiscreening involves the simultaneous screening for multiple diseases using integrated diagnostic methods, potentially improving early detection rates and optimizing resource utilization. In 2024, Rzyman W. et al. launched the MULTIPREVENT epidemiological study, which aims to develop and validate a low-dose computed tomography (LDCT)-based screening test for civilizational diseases. This study represents a step forward in the pursuit of more effective, minimally invasive diagnostic tools that could facilitate earlier intervention and improve patient outcomes. To better understand the potential of multiscreening approaches and their clinical utility, it is essential to evaluate the existing predictive models used for identifying individuals at high risk for these diseases. This narrative review focuses primarily on lung cancer risk prediction models used in LDCT screening while situating these approaches within the broader conceptual framework of the MULTIPREVENT project, aimed at future integration of multi-disease prevention strategies. With this analysis, we aim to provide insights that will guide the development of more accurate, integrative screening tools that could reduce the global burden of these diseases.

## 1. Introduction

Lung cancer remains the leading cause of cancer-related mortality worldwide, with late-stage diagnosis contributing significantly to its high fatality rate [1]. Alongside other prevalent chronic conditions, such as cardiovascular disease, diabetes, and chronic obstructive pulmonary disease, lung cancer poses a major public health challenge. Early detection through low-dose computed tomography screening offers substantial potential to reduce mortality by identifying the disease at a treatable stage [2].

The project titled “Multimorbidity—personalized preventive healthcare for adults” (acronym MULTIPREVENT; Medical Research Agency 2023/ABM/03/00036), initiated by the Medical University of Gdańsk, was developed in response to the limitations of conventional screening programs, which typically focus solely on age and smoking history, often neglecting other significant risk factors. MULTIPREVENT introduces a more personalized approach aimed at enhancing risk stratification and improving outreach to underrepresented populations, particularly individuals affected by multimorbidities [3].

One of the key areas where such personalized prevention can bring significant benefits is lung cancer screening using low-dose computed tomography (LDCT). LDCT has proven to be effective in detecting cancer at earlier, more treatable stages and in reducing mortality [4]. However, it also carries certain risks. These include false-positive results, which may lead to unnecessary medical procedures, increased patient anxiety, and higher healthcare costs. Participation in screening programs is also hindered by factors such as stigma, fear of diagnosis, and the presence of comorbidities [2]. These challenges underscore the need for personalized strategies to enhance patient engagement.

This publication focuses exclusively on lung cancer screening, providing a critical review of both eligibility criteria and risk-based predictive models. It evaluates their strengths and limitations in identifying high-risk populations, discusses key principles of screening, and synthesizes existing evidence. The analysis is situated within the context of the MULTIPREVENT project—an epidemiological study led by the Medical University of Gdańsk—which aims to develop an integrative diagnostic framework capable of identifying early markers, risk profiles, and predictive features associated with the onset and progression of the four most common lifestyle-related diseases: lung cancer, cardiovascular disease, diabetes, and chronic obstructive pulmonary disease. Participants of the MOLTEST-BIS lung cancer screening project (2016–2018) are invited to take part in the MULTIPREVENT study [3].

## 2. Materials and Methods

This study was designed as a narrative review of the literature. A literature search was conducted using the PubMed database (U.S. National Library of Medicine, Bethesda, MD, USA). The last search was performed on 8 November 2025. The analysis focused on publications published within the last 10 years.

Search terms included combinations of the following keywords: “lung cancer screening”, “low-dose computed tomography”, “risk prediction models”, and “early-stage cancer detection”.

The search was limited to articles published in English and Polish. Priority was given to landmark randomized controlled trials, validation studies of lung cancer risk prediction models, and relevant review articles. Additional references were identified through manual screening of reference lists from key publications and current clinical guidelines.

Given the narrative nature of this review, no formal inclusion or exclusion criteria or PRISMA workflow were applied.

## 3. Risk Prediction Models

Effective identification of individuals at elevated risk for lung cancer is essential for improving the outcomes of screening programs.

Classical predictive models primarily rely on simple, predefined criteria such as age thresholds and cumulative smoking exposure (e.g., pack-years). These models, exemplified by the National Lung Screening Trial (NLST) and the Dutch–Belgian Cancer Screening Trial (NELSON), are valued for their simplicity and ease of application in large populations. However, due to their restrictive criteria, they often lack sensitivity in detecting risk among certain high-risk populations, including former smokers, women, and individuals with multiple comorbidities [5,6].

In contrast, risk-based predictive models utilize multivariable algorithms that integrate a broader spectrum of factors, including demographic, clinical, and behavioral data, to estimate an individual’s lung cancer risk within a specific time frame. Models such as the Prostate, Lung, Colorectal and Ovarian Cancer Screening Trial Model 2012 (PLCOm2012), the Bach model, and the Liverpool Lung Project (LLP) model have demonstrated superior performance in identifying high-risk individuals and encompass a more diverse patient population than classical models. These approaches offer a more refined and personalized risk assessment [7]. To further elaborate on these approaches, the subsequent sections provide a comprehensive examination of selected individual predictive models, highlighting their design, advantages, and limitations. The key characteristics of the described prediction models are summarized in Table 1.

### 3.1. Classical Predictive Models

#### 3.1.1. NLST—National Lung Screening Trial

The National Lung Screening Trial is one of the earliest and most widely recognized examples of a classical predictive model, and it has played a foundational role in shaping current lung cancer screening guidelines. As a classical model, it is based on a limited set of easily measurable criteria, without incorporating more complex individual risk factors or multivariable analysis. Its simplicity facilitated large-scale implementation, but also contributed to limited sensitivity in detecting risk among certain subpopulations [8].

The inclusion criteria for the NLST were: age between 55 and 75 years, a smoking history of at least 30 pack-years, and, for former smokers, cessation within the past 10 years [9]. Consequently, these criteria may fail to identify a significant number of individuals who will eventually develop lung cancer, and may lack sensitivity in selecting true cases within screening populations.

Nevertheless, application of this model demonstrated that low-dose computed tomography (LDCT) screening resulted in 20% reduction in lung cancer mortality [2,8].

#### 3.1.2. NELSON—Dutch–Belgian Lung Cancer Screening Trial (NEderlands–LEuvens Longkanker Screenings ONderzoek)

Another example of a classical predictive model is the Dutch–Belgian Lung Cancer Screening Trial (NELSON). Similar to the NLST, the NELSON trial relied on relatively simple inclusion criteria and did not incorporate individualized risk estimation. The inclusion criteria in NELSON were similar in structure to those used in the NLST but differed slightly in specific thresholds. Participants were eligible if they were between 50 and 74 years of age, had smoked at least 15 cigarettes per day for 25 years or at least 10 cigarettes per day for over 30 years, and, in the case of former smokers, had quit less than 10 years prior to enrollment [9]. Despite relying on relatively simple eligibility criteria, this predictive model also demonstrated clinical utility, reporting a 24% reduction in lung cancer mortality in the LDCT group compared to no screening [9].

### 3.2. Risk-Based Predictive Models

#### 3.2.1. PLCOm2012—Prostate, Lung, Colorectal, and Ovarian Cancer Screening Trial Model 2012

This model was developed using data from the PLCO Screening Trial, one of the largest randomized controlled trials evaluating cancer screening effectiveness. It was designed within the context of a multicancer screening initiative, but it also estimates the individualized 6-year risk of developing lung cancer. It incorporates a set of variables, including age, sex, race/ethnicity, education level, BMI, smoking status and intensity (pack-years), years since smoking cessation, personal history of COPD, personal and family history of cancer and occupational exposures [7].

Validation in the Polish MOLTEST-BIS cohort (n = 6631) included participants aged 50–79 years with smoking history of at least 30 pack-years. The study applied a high-risk threshold of ≥1.3% six-year risk according to the PLCOm2012 model. Results showed that PLCOm2012 identified 97.4% of lung cancer cases with an AUC of 0.717, qualifying 82.4% of participants for LDCT screening. This high sensitivity, achieved through incorporating multifactorial risk elements, makes this model an effective and inclusive risk stratification tool, as it minimizes missing lung cancer cases [7].

#### 3.2.2. Bach Model

The Bach model is a simplified risk prediction tool developed based on epidemiological studies. It estimates an individual’s 5-year risk of developing lung cancer, with primary targeting populations with a history of smoking. The model incorporates variables such as age, sex, cumulative smoking intensity (measured in pack-years), duration of smoking, time since cessation, and exposure to asbestos [7].

Compared to the PLCOm2012 model, the Bach model has a narrower scope, considering fewer risk factors. It focuses mainly on basic demographic data and smoking history, while excluding clinical and socio-economic variables. This limited approach results in lower sensitivity and a higher likelihood of missing lung cancer cases [7].

In the MOLTEST-BIS study, the Bach model identified only 44.8% of lung cancer cases and recommended screening for 19.8% of participants. Its AUC was 0.701, reflecting moderate predictive performance. Although the model is operationally straightforward and facilitates quick assessment, its restricted range of considered factors may lead to the omission of a significant number of high-risk individuals, thereby limiting its effectiveness in lung cancer screening programs [7].

#### 3.2.3. LLP—Liverpool Lung Project Model

This model was developed as part of a large-scale epidemiological study conducted in the UK, aimed at stratifying lung cancer risk. It estimates a 5-year risk of developing lung cancer by integrating demographic, clinical and environmental factors. It includes variables such as age, smoking duration, personal and family history of lung cancer, history of pneumonia, and occupational exposure to asbestos [7].

In the MOLTEST-BIS study population, the LLP model detected 74.0% of lung cancer cases and qualified 50.3% of participants for screening, achieving an AUC of 0.667—the lowest among the three models validated in this study. Although its sensitivity is lower than PLCOm2012 model, LLP remains a practical option due to its moderate complexity, making it suitable for routine clinical use [7].

Among the evaluated models, PLCOm2012 appears to be the most suitable for implementation within the multiscreening framework, owing to its high sensitivity, inclusion of comorbidity-related variables, and validated performance in European populations. While simpler models such as Bach or LLP offer operational ease, their lower sensitivity may limit their utility in comprehensive preventive strategies aimed at addressing multimorbidity.

### 3.3. Using Biomarkers—The Integrative Analysis of Lung Cancer Etiology and Risk Program (INTEGRAL)

The Integrative Analysis of Lung Cancer Etiology and Risk (INTEGRAL) program is an initiative aimed at improving the effectiveness of lung cancer screening by integrating blood-based protein biomarkers with existing risk assessment models. Supported by the National Cancer Institute, the project consists of two main parts: the Risk Biomarker project, which focuses on identifying and validating biomarkers for preselecting high-risk individuals, and the Nodule Malignancy project, which aims to differentiate benign from malignant lung nodules detected during screening [10].

Using proteomic technologies, researchers from this project analyzed over 1100 proteins in blood samples from more than 1600 lung cancer cases. Then, they compared these results with matched control groups from multiple cohorts to identify cancer-related biomarkers. They refined this to a panel of 21 proteins that demonstrated high accuracy in predicting short-term lung cancer risk, with AUC reaching up to 0.90. Furthermore, the Nodule Malignancy project successfully applied these biomarkers to distinguish malignant nodules from benign ones in over 1400 participants undergoing LDCT [10].

These findings suggest that including protein biomarkers in lung cancer risk assessment may improve the accuracy of screening eligibility and reduce the number of unnecessary procedures. However, it has been emphasized that further large-scale validation studies are needed before such an approach can be implemented in clinical practice [10].

In conclusion, the future of effective lung cancer screening should combine traditional epidemiological risk factors with molecular biomarkers. This approach could help with optimizing early cancer detection and maximizing patient benefits.

## 4. Risk Group

For any lung cancer screening program to be truly effective and generate meaningful outcomes, the foundation must lie in appropriately defined inclusion criteria and accurate participant selection. Identifying individuals at the highest risk is essential not only for maximizing the clinical benefits of early detection but also for ensuring the efficient use of healthcare resources. As demonstrated by the predictive models discussed earlier, various approaches can be employed to determine eligibility, ranging from classical models based on simple, predefined thresholds to more complex, risk-based algorithms incorporating multiple variables. Each method presents distinct advantages and limitations, particularly in terms of sensitivity, inclusiveness, and feasibility of implementation. Regardless of the specific model applied, several recurring factors shape the definition of the target population for lung cancer screening. These include basic demographic characteristics (such as age and sex), smoking history (including intensity, duration, and time since cessation), and, in more advanced models, clinical history, comorbidities, family history of cancer, and environmental exposures. Taken together, these variables form the foundation for identifying individuals at increased risk and determining who may benefit most from participation in screening programs [7].

However, defining appropriate eligibility criteria is only the first step toward building an effective screening program. Equally important, and often more challenging, is ensuring that these criteria are applied in a way that successfully reaches the intended population. Attention must be given to engaging individuals from so-called hard-to-reach groups, who may meet clinical inclusion criteria but remain underrepresented due to socioeconomic, cultural, psychological, or logistical barriers. Research shows that certain high-risk individuals are systematically less likely to participate in CT screening despite meeting eligibility requirements. These include older adults, women, current smokers, individuals from lower socioeconomic backgrounds, and those with heightened emotional or affective risk perception. In contrast, former smokers tend to respond more positively to screening invitations, likely due to their perceived proactive stance toward health following cessation. Notably, even within high-risk populations, participation patterns vary; high-risk women are generally less likely to take part in screening than their male counterparts. Interestingly, a personal history of chronic lung disease has been associated with a greater willingness to undergo chest LDCT, possibly due to increased perceived vulnerability and heightened health awareness [11].

Addressing the health needs of high-risk populations, particularly those facing multiple overlapping risk factors, requires more than just improved recruitment strategies. It also calls for a deeper understanding of their broader clinical profiles and comorbidities. In this context, the MULTIPREVENT project provides an important framework for investigating how tobacco exposure, age, genetic background, and respiratory disease history jointly influence lung cancer risk [3]. Building on data from 3000 participants previously enrolled in the MOLTEST-BIS screening program, the study aims not only to refine risk stratification but also to explore coexisting conditions common in this population, such as COPD, cardiovascular disease, and diabetes [7]. Although the primary focus remains lung cancer, the project’s integrative approach combining LDCT with additional tests underscores the need for coordinated, multi-disease prevention strategies. This is particularly relevant for individuals from socioeconomically disadvantaged or medically underserved backgrounds, where the cumulative burden of disease often remains unrecognized and undertreated. Beyond its established role in lung cancer screening, LDCT of the chest may serve as a powerful tool for the early detection of multiple conditions, including other thoracic malignancies, cardiovascular disease, emphysema, osteoporosis, hepatic steatosis, and pulmonary fibrosis. Integrating LDCT findings with simple complementary assessments such as HbA1c measurement (blood test), spirometry, body composition analysis, and blood pressure monitoring could enable not only the simultaneous identification but also the prediction of the major causes of mortality in developed countries: cancer, COPD, cardiovascular disease, and diabetes. The translational impact of such findings could represent an important first step toward entirely new, coordinated strategies for the prevention of cancer and other major chronic diseases [3].

## 5. Main Obstacles in Recruitment

The success of screening programs depends heavily on the recruitment strategies employed. As previously noted, rigid, classical inclusion criteria, based solely on age and smoking history, as used in trials such as the NLST, systematically exclude subgroups such as women, former smokers, and individuals with multiple chronic conditions. This limitation reduces the population-level impact of screening and highlights the need for more inclusive methods of participant identification.

Insights from the Cancer Care Ontario (CCO) pilot study illustrate the crucial role of recruitment strategies in overcoming these challenges. Conducted across three centers and enrolling 3294 participants, the CCO program demonstrated the critical role of primary care physician referrals in lung cancer screening recruitment effectiveness. Over 80% of participants were enrolled through direct referrals from trusted family doctors, emphasizing how personalized physician engagement substantially outperforms other recruitment methods such as mass mailings or media campaigns, which often yielded response rates below 1% and incurred disproportionately high costs [12]. This trend is further supported by findings from the UK Lung Cancer Screening (ULKS) Trial, where invitations sent directly by general practitioners achieved a response rate of approximately 26%, compared to the 0.2–3.7% typically observed in the US [12].

The importance of physician involvement is further proven by the experience of the U.S. Veterans Affairs program, which relied on automated identification through electronic medical records (EMRs). Although the system flagged eligible individuals based on age and smoking history, only 28% were subsequently reviewed by a physician, and of those, only half were screened. This shows a key limitation of system-based approaches when not paired with active clinical engagement [12].

Beyond recruitment methods themselves, the use of detailed participant registries that combine clinical data, lifestyle information, and responses to individual questionnaires facilitates identification of high-risk individuals, especially those who might be overlooked by standard inclusion criteria. The MULTIPREVENT project exemplifies this approach by integrating risk prediction tools (PLCOm2012 in MOLTEST-BIS) with comprehensive databases, enabling outreach to individuals with complex health needs, such as those living with COPD or multiple chronic conditions [3].

Nonetheless, psychological and social barriers remain significant obstacles to screening participation. Feelings of guilt associated with smoking, fear of diagnosis, and general mistrust of the healthcare system are particularly prevalent among communities that have experienced historical discrimination or among individuals with lower socioeconomic status (SES) and limited health literacy. To address these challenges, culturally sensitive approaches are essential. These include engaging local communities and involving peer recruiters who share lived experiences or backgrounds with the target population [12].

Taken together, the results from the CCO, ULKS and U.S. Veterans Affairs programs demonstrate that personal invitations from trusted healthcare providers are significantly more effective than impersonal methods like mass mailings or algorithm-based identification alone. Physician-led recruitment not only improves response rates but also helps overcome psychological and social barriers, especially among individuals with lower health literacy or socioeconomic disadvantage [12].

However, despite the overall effectiveness of physician-led recruitment strategies, not all population groups are equally represented in lung cancer screening programs. Persistent gaps in participation suggest that additional barriers may exist beyond the recruitment method itself. Key factors associated with underrepresentation in current research include low socioeconomic status, current smoking, female sex (with fewer women than men participating in Lung Cancer Screening—LCS), and a family history of lung cancer or exposure to carcinogenic factors [11]. Among the various factors influencing patients’ decisions to participate in lung cancer screening, four appear to be particularly impactful. One of the strongest motivators is the belief in the benefits of early detection, especially when patients understand that identifying cancer at an earlier stage can significantly improve treatment outcomes. This is further reinforced by the perception that LDCT LCS involves minimal risks or side effects, making it a relatively low-burden procedure. Emotional drivers also play a role—individuals who have witnessed family members or close friends face late-stage cancer often develop a heightened sense of urgency and personal relevance. Ultimately, however, the most decisive factor is a trusted physician’s recommendation, which consistently proves to be the strongest catalyst for participation [11].

**Figure 1:**
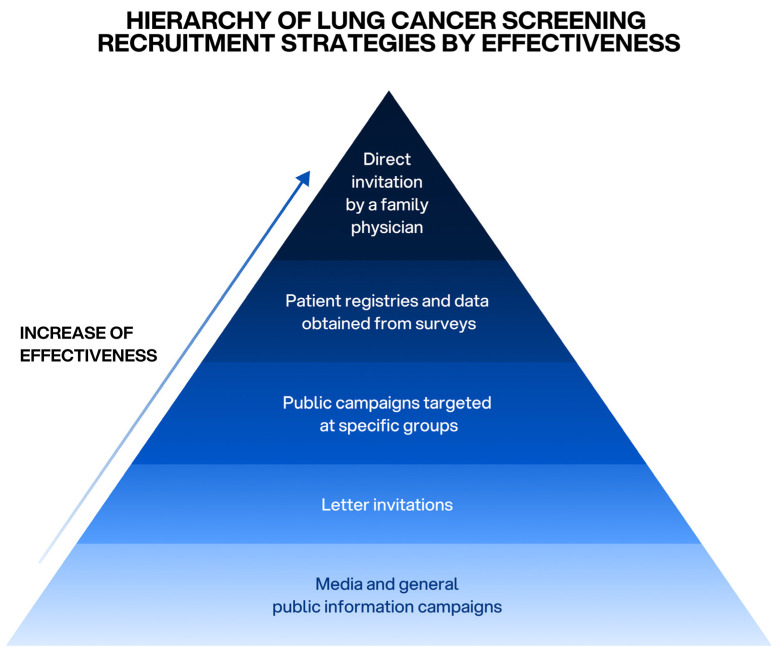
Pyramid illustrating recruitment strategies in lung cancer screening arranged by increasing effectiveness. The base of the pyramid represents broad, population-level approaches aimed at large, heterogeneous audiences, characterized by minimal personalization. As recruitment strategies move upward, they become progressively more individualized and targeted, focusing on identified high-risk individuals. This increasing degree of personalization is associated with greater engagement and higher recruitment effectiveness, culminating in clinician-mediated, person-centered invitations at the apex [12].

A summary of the relative effectiveness of patient recruitment strategies is presented in Figure 1.

## 6. Harms and Benefits of Participation

A key element in the development and implementation of any screening program is the careful assessment of the balance between potential benefits and possible harms. While early detection through LDCT screening has been shown to significantly reduce lung cancer mortality, screening is not without risks [13]. False positives, overdiagnosis, radiation exposure, and psychological distress are important concerns that must be weighed against the life-saving potential of early intervention [14].

Among the potential harms associated with participation in lung cancer screening programs, radiation exposure from LDCT is often the first to be considered. Although unavoidable in CT imaging, radiation carries a potential risk of inducing malignancies over time, particularly with repeated exposure [15]. While this concern is legitimate, the actual magnitude of radiation-induced cancer risk from LDCT screening remains relatively low, especially when compared to the demonstrated reduction in mortality. In the COSMOS study, which followed over 5000 participants undergoing annual LDCT screening for 10 years, the estimated additional risk of major cancer was only 0.05%, corresponding to approximately one radiation-induced cancer for every 108 lung cancers detected [16]. These findings suggest that the benefits of early detection significantly outweigh the risks associated with radiation exposure, particularly in high-risk populations. Thus, although not negligible, radiation risks are considered acceptable within well-designed screening programs.

Another important drawback of lung cancer screening is the emotional burden it may impose on participants. This psychological impact often stems from false-positive results, which can lead to unnecessary anxiety, additional diagnostic procedures, and temporary uncertainty regarding one’s health status [14]. Moreover, many individuals enter screening programs feeling asymptomatic and healthy, making the possibility of an unexpected abnormal finding particularly distressing. Even short-term anxiety should not be underestimated when evaluating the overall harms and benefits of screening. In the NLST, low-dose CT screening led to at least one positive finding in 39.1% of participants, with 96.4% of those ultimately proven to be false positives over three screening rounds [2]. These data underscore the importance of acknowledging and addressing the psychological dimension of lung cancer screening. While the clinical benefits of early detection are well established, the emotional consequences of false alarms and diagnostic uncertainty must not be overlooked. Comprehensive screening programs should therefore incorporate not only technical accuracy and clinical outcomes but also measures to support participants’ emotional well-being.

A further concern is the risk of overdiagnosis—an inherent limitation of many screening programs. Overdiagnosis refers to the detection of cancers that, if left undetected and untreated, would never have caused symptoms or led to death during a patient’s lifetime. Although often indistinguishable from clinically significant tumors at the time of diagnosis, these findings may lead to unnecessary interventions, exposing patients to potential harm without therapeutic benefit, a phenomenon known as overtreatment [17]. An analysis of overdiagnosis in the NLST estimated that for every 320 individuals screened to prevent one lung cancer death, approximately 1.38 cases of overdiagnosis would occur [18]. These findings highlight the need to explicitly acknowledge overdiagnosis as a potential risk associated with LDCT screening and to communicate this clearly during patient outreach and recruitment.

Shared decision-making is a crucial component of effective cancer screening, including lung cancer screening. For patients to make informed choices, they must be provided with clear, balanced information about both the potential benefits and the possible risks of participation. However, achieving this standard can be challenging due to gaps in provider knowledge and communication. When healthcare professionals lack a comprehensive understanding of screening protocols or fail to convey the full scope of relevant information, patients may not receive the guidance necessary to make truly informed decisions. Therefore, ensuring that individuals are properly informed and actively involved in the decision to participate is essential to maintaining the integrity and ethical foundation of screening programs.

In the case of the MULTIPREVENT project, the study has been designed to address not only the clinical aspects of lung cancer screening but also the psychological needs of participants. The program includes a dedicated consultation with a pulmonologist after the examination to discuss individual results and potential next steps in diagnosis or management. This structured interaction represents an important component of the screening process, allowing participants to better understand the meaning of their results and the broader context of their health status. The opportunity to openly discuss the procedure, the outcomes, and possible future scenarios can significantly reduce anxiety and emotional distress associated with screening participation, particularly in cases of indeterminate or positive findings. In this way, MULTIPREVENT responds to the psychological dimension of screening, supporting participants not only medically but also emotionally [3].

## 7. Discussion

### 7.1. Evolution of Risk-Adapted Lung Cancer Screening

Lung cancer screening has entered a stage of methodological (re)evolution, moving beyond simple demographic criteria toward more risk-adapted and integrative approaches. In line with the aim of this analysis, which is to provide insights guiding the development of more accurate and comprehensive screening tools capable of reducing the global burden of chronic diseases, our findings highlight the central role of validated prediction models such as PLCOm2012 and the need for their integration into broader preventive frameworks like MULTIPREVENT. These initiatives exemplify how predictive modeling, personalized recruitment, and multi-disease assessment can converge to enhance both the accuracy and the public health impact of screening programs.

### 7.2. Performance of Validated Risk Prediction Models: Focus on PLCOm2012

Among the currently validated models, the PLCOm2012 model has demonstrated the highest sensitivity for identifying individuals at elevated risk of lung cancer. Unlike traditional approaches, it incorporates variables such as education level, race, history of chronic obstructive pulmonary disease, and family history of lung cancer, which collectively improve its discriminative ability. The results obtained within the MOLTEST-BIS study confirm that PLCOm2012 performs well in European populations and can be successfully applied in different healthcare systems. Nevertheless, further investigation is needed to improve its adjustment and practical usability, ensuring that its implementation is both effective and resource efficient.

### 7.3. Biomarker-Enhanced Risk Stratification

A major direction for future development involves the integration of risk models with molecular and biomarker data, which may significantly enhance screening precision. The INTEGRAL program exemplifies such innovation, combining proteomic and genomic information with traditional risk factors to refine risk estimation and improve early detection. These hybrid approaches show promise in identifying individuals who might otherwise remain outside current screening criteria, pointing toward a new generation of precision screening strategies.

### 7.4. Recruitment and Participation in High-Risk Groups

Despite advances in risk modeling, the overall success of screening programs depends not only on predictive accuracy but also on recruitment strategies and population engagement. Reaching those who are most at risk remains a challenge, particularly among women, active smokers, individuals with multiple comorbidities, and those with lower socioeconomic status groups that are often underrepresented in screening cohorts. Evidence indicates that physician-led, personalized recruitment is more effective than mass invitations, as it fosters trust, ensures better understanding of the process, and promotes shared decision-making. Reflecting this insight, MULTIPREVENT actively involves healthcare providers and utilizes personalized invitations to encourage participation among high-risk individuals from MOLTEST-BIS.

### 7.5. Integrated Multidisease Prevention in MULTIPREVENT

Beyond methodological innovation, MULTIPREVENT represents a comprehensive, integrative approach to preventive medicine. It is based on the combination of predictive modeling and risk-based recruitment performed in MOLTEST-BIS with the simultaneous assessment of multiple chronic diseases, including lung cancer, cardiovascular, and metabolic conditions. This multi-disease screening framework exemplifies how prevention can move toward efficiency and a patient-centric approach. Moreover, MULTIPREVENT addresses the psychological dimension of screening participation, which is a frequently neglected aspect in large-scale programs. Participation in the project begins with an interview, during which participants discuss their health status, ask questions, and receive clear information about the procedures and their purpose. The project also includes a post-screening consultation with a pulmonologist, providing participants with an opportunity to discuss their results, clarify uncertainties, and consider next steps. This structured communication has the potential to mitigate anxiety, enhance understanding, and promote long-term engagement with preventive care.

Within the MULTIPREVENT project, LDCT-based lung cancer screening constitutes the primary component of the study. MOLTES-BIS participant risk stratification was supported by validated lung cancer prediction models, such as PLCOm2012, which was used to identify individuals at increased risk who were most likely to benefit from screening. Now they are invited to continue screening within broader concept of MULTIPREVENT. Data derived from MOLTEST-BIS program provide an empirical basis for evaluating and refining this approach.

At the operational level, the current workflow focuses on LDCT imaging and structured clinical interpretation of imaging findings; however, MULTIPREVENT evaluates a broader concept of risk stratification, considering multimorbidity. Conceptually, this approach integrates LDCT results with selected, readily available clinical measures (e.g., spirometry, HbA1c, blood pressure, body composition) and disease-specific risk scores for COPD, cardiovascular disease, and diabetes. Importantly, this integrated approach based on multiscreening provides a future direction for preventive populational screening strategies.

## 8. Conclusions

In conclusion, effective prevention strategies depend on the personalized and integrated combination of molecular, clinical, and communicative components within a unified framework. Projects such as MULTIPREVENT exemplify how validated predictive models, proactive recruitment, and psychosocial support can act synergistically to enhance benefits of screening while mitigating potential harms. Future research should prioritize the optimization of these integrative approaches and their translation into population-based settings to achieve sustainable improvements in public health and cancer prevention outcomes.

## Figures and Tables

**Table 1:** Comparison of classical and risk-based prediction models used for lung cancer screening.

Criterion	NLST	NELSON	PLCOm2012	Bach	LLP
**Approach**	Not risk-based model	Not risk-based model	Risk-based model	Risk-based model	Risk-based model
**Risk horizon**	Not applicable	Not applicable	6 years	5 years	5 years
**Key prognostic factors**	Age, pack-years, smoking status	Age, smoking intensity and duration	Age, ethnicity, education, body mass index, family history of lung cancer, personal history of cancer, COPD diagnosis, smoking status, smoking duration, number of cigarettes per day, time elapsed since quitting	Age, gender, asbestos exposure, smoking intensity, smoking duration, quit time in former smokers	Age, presence of pneumonia, asbestos exposure, personal history of cancer, family history of cancer, smoking duration
**AUC**	Not applicable	Not applicable	0.717 ^1^	0.701 ^1^	0.667 ^1^
**Sensitivity-specificity trade-offs**	High specificity, lower sensitivity (excludes subgroups)	Improved specificity via nodule volumetry	Highest sensitivity at practical specificity (97.4% cases, 82.4% eligible)	Lowest sensitivity, highest specificity (44.8% cases, 19.8% eligible)	Moderate sensitivity/specificity balance (74% cases, 50.3% eligible)
**Proportion of population eligible**	~26% of ever-smokers ^2^	No published estimate (but criteria broader than NLST)	82.4% ^1^	19.8% ^1^	50.3% ^1^
**Calibration**	Not applicable (criteria-based)	Not applicable (criteria-based)	Good external validation (MOLTEST-BIS AUC 0.717, well-calibrated)	Limited external calibration (overestimates in some EU cohorts, AUC 0.701)	Population-specific calibration required (lowest AUC 0.667, needs recalibration)
**Implementation complexity**	Low	Moderate	Moderate	Low–moderate	Moderate
**System-level costs**	Lower upfront, inefficient targeting	Moderate, optimized follow-up	More efficient resource use	Lower screening volume	Higher screening and follow-up burden
**Main strengths**	Simplicity; mortality reduction	Reduced false positives	Highest sensitivity (97.4%), inclusive risk stratification	Parsimonious (simple variables), fast implementation	Includes genetic/environmental factors
**Main limitations**	Binary eligibility miss subgroups	No individualized risk	High screening volume (82.4%), data-intensive	Lowest sensitivity (44.8%)	Lowest AUC (0.667)

^1^ The data were derived from the MOLTEST-BIS project [7]. ^2^ The data were derived from the source numbered [8].

## Data Availability

No new data were created or analyzed in this study.

## Abbreviations

The following abbreviations are used in this manuscript:

LC	Lung cancer
CVD	Cardiovascular diseases
COPD	Chronic obstructive pulmonary disease
LDCT	Low-dose computed tomography
NLST	National Lung Screening Trial
NELSON	NEderlands–LEuvens Longkanker Screenings ONderzoek
PLCOm2012	Prostate, Lung, Colorectal, and Ovarian Cancer Screening Trial Model 2012
LLP	Liverpool Lung Project (model)
INTEGRAL	Integrative Analysis of Lung Cancer Etiology and Risk
AUC	Area Under the Curve
HbA1c	Hemoglobin A1c blood test
CCO	Cancer Care Ontario
ULKS	UK Lung Cancer Screening (Trial)
EMRs	Electronic Medical Records
SES	Socioeconomic status
LCS	Lung Cancer Screening
